# Novel TRPV6 mutations in the spectrum of transient neonatal hyperparathyroidism

**DOI:** 10.1186/s12576-020-00761-2

**Published:** 2020-07-09

**Authors:** Yoshiro Suzuki, Hirotake Sawada, Tomoko Tokumasu, Shigeru Suzuki, Shinsuke Ninomiya, Masaru Shirai, Tokuo Mukai, Claire T. Saito, Gen Nishimura, Makoto Tominaga

**Affiliations:** 1 Department of Physiology, Iwate Medical University, 1-1-1 Idaidori, Yahaba-cho, Shiwa-gun, Iwate, 028-3694 Japan; 2 Division of Cell Signaling, National Institute for Physiological Sciences (Exploratory Research Center on Life and Living Systems), National Institutes of Natural Sciences, Okazaki, 444-8787 Japan; 3 Department of Physiological Sciences, SOKENDAI (The Graduate University for Advanced Studies), Okazaki, 444-8787 Japan; 4 Department of Fundamental Nursing, Faculty of Medicine, University of Miyazaki, Miyazaki, 889-1692 Japan; 5 Department of Pediatrics, Kurashiki Central Hospital, Kurashiki, 710-8602 Japan; 6 Department of Pediatrics, Asahikawa-Kosei General Hospital, Asahikawa, 078-8211 Japan; 7 Department of Pediatrics, Asahikawa Medical University, Asahikawa, 078-8510 Japan; 8 Department of Clinical Genetics, Kurashiki Central Hospital, Kurashiki, 710-8602 Japan; 9 Department of Pediatrics, Japanese Red Cross Asahikawa Hospital, Asahikawa, 070-8530 Japan; 10 Center for Intractable Disease, Saitama Medical University Hospital, Saitama, 350-0495 Japan

**Keywords:** Transient receptor potential channel (TRP channel), Maternal–fetal calcium transport, Transient neonatal hyperparathyroidism (TNHP), Patch-clamp recording, Membrane trafficking

## Abstract

Maternal–fetal calcium (Ca^2+^) transport in the placenta plays a critical role in maintaining fetal bone mineralization. Mutations in the gene encoding the transient receptor potential cation channel, subfamily V, member 6 (TRPV6) have been identified as causative mutations of transient neonatal hyperparathyroidism due to insufficient maternal–fetal Ca^2+^ transport in the placenta. In this study, we found two novel mutations in subjects that have transient neonatal hyperparathyroidism. TRPV6 carrying the mutation p.Arg390His that localizes to the outer edge of the first transmembrane domain (S1) showed impaired trafficking to the plasma membrane, whereas TRPV6 having the mutation p.Gly291Ser in the sixth ankyrin repeat (AR) domain had channel properties that were comparable those of WT channels, although the increases in steady-state intracellular Ca^2+^ concentration could have led to Ca^2+^ overload and subsequent death of cells expressing this mutant channel. These results indicate that the AR6 domain contributes to TRPV6-mediated maintenance of intracellular Ca^2+^ concentrations, and that this region could play a novel role in regulating the activity of TRPV6 Ca^2+^-selective channels.

## Background

Calcium (Ca^2+^) is essential for many physiological functions. Blood Ca^2+^ levels are finely maintained by the parathyroid hormone (PTH)–vitamin D axis that modulates bone resorption and intestinal Ca^2+^ absorption. In fetuses and neonates, additional Ca^2+^ transport occurs to maintain bone mineralization [1–3]. In many mammalian species, including humans, fetal blood Ca^2+^ levels are higher than those in mature individuals, suggesting that the transport is mediated by uphill or energy-consuming transport through epithelial, transcellular, rather than paracellular routes [4, 5]. Ca^2+^ via the transcellular pathway is achieved via three stages: (1) Ca^2+^ uptake through a channel driven by an electrochemical Ca^2+^ gradient; (2) binding to calbindin that does not increase free calcium concentrations, and (3) basolateral extrusion of Ca^2+^ by plasma membrane Ca^2+^-ATPase or Na^+^/Ca^2+^-exchangers [6, 7]. However, the proteins involved in regulating this pathway and the associated regulatory mechanisms for maternal–fetal calcium transport remain unclear.

Transient neonatal hyperparathyroidism is a neonatal bone disorder caused by insufficient fetal bone mineralization due to impaired maternal–fetal Ca^2+^ transport across the placenta. Recently, mutations in the gene encoding transient receptor potential cation channel, subfamily V, member 6 (TRPV6) were identified as causative mutations of transient neonatal hyperparathyroidism (TNHP) [8]. There are several hotspots for TRPV6 mutations: the outer edges of the transmembrane domain S2 and S3 that affect trafficking to the plasma membrane; the fourth ankyrin repeat (AR) domain that affects protein stability; and the intracellular S2–S3 loop that affects intracellular Ca^2+^-dependent inactivation. The different mutations suggest that there are several mechanisms for TNHP disease onset. Moreover, a combination of mutations could also be important for disease pathogenesis since many patients carry compound heterozygous mutations. Here we report novel mutations in the S1 transmembrane domain and the sixth AR domain. The functional significance of these mutations revealed an unexpected role for the AR domain in TRPV6 activity.

## Methods

### Subjects

*Subject 1* This male patient was born to a 39-year-old G2P1 mother of Japanese descent; the father was 44 years old and also of Japanese descent. The couple was non-consanguineous. They had an older daughter who had shorter limbs at 8 months of gestation, but is currently normal. The father had a funnel chest and shorter limbs and there was a family history of similar physical features. Genetic information for the parents and other family members was not available.

Fetal ultrasound at 29 weeks of gestation showed polyhydramnios and thoracic narrowing with rib deformities. Although the mother was treated with uterine contraction inhibitors to prevent premature delivery, uterine contractions began at 36 weeks of gestation and the child was born by a scheduled cesarean section the day after contractions began. The birth weight was 2.351 kg and the length was 41 cm. The occipitofrontal circumference (OFC) was 34.8 cm. Apgar scores were 4, 5, 8 at 1, 5, 10 min, respectively. Artificial invasive ventilation was initiated on day 0. On day 8, the child was taken off the ventilator, but retractive breathing and hyperpnea occurred with normal SpO_2_ level. Therefore, neutrally adjusted ventilatory assist was applied on the same day as ventilator removal.

Radiographs showed a narrow thorax, femoral shortening and lower bone mineral density with ossification failure, which was suggestive of hyperparathyroidism. Cord blood levels of intact PTH (iPTH) were extremely high (high sensitive PTH was > 3200 pg/ml and iPTH was 1371 pg/ml), which, together with lower than normal levels of Ca^2+^ and vitamin D and maternal hypovitaminosis D, led to a diagnosis of hyperparathyroidism secondary to maternal vitamin D deficiency. I-cell disease was not likely since lysosomal enzyme activity was normal. The subjected was treated with vitamin D, Ca, and P, which improved iPTH, alkaline phosphatase (ALP), Ca, P, and vitamin D levels. Thorax and bone mineral density was also improved by this treatment. After removing the ventilator, the noninvasive positive pressure ventilator was used until the child reached 13 months of age.

**Fig. 1:**
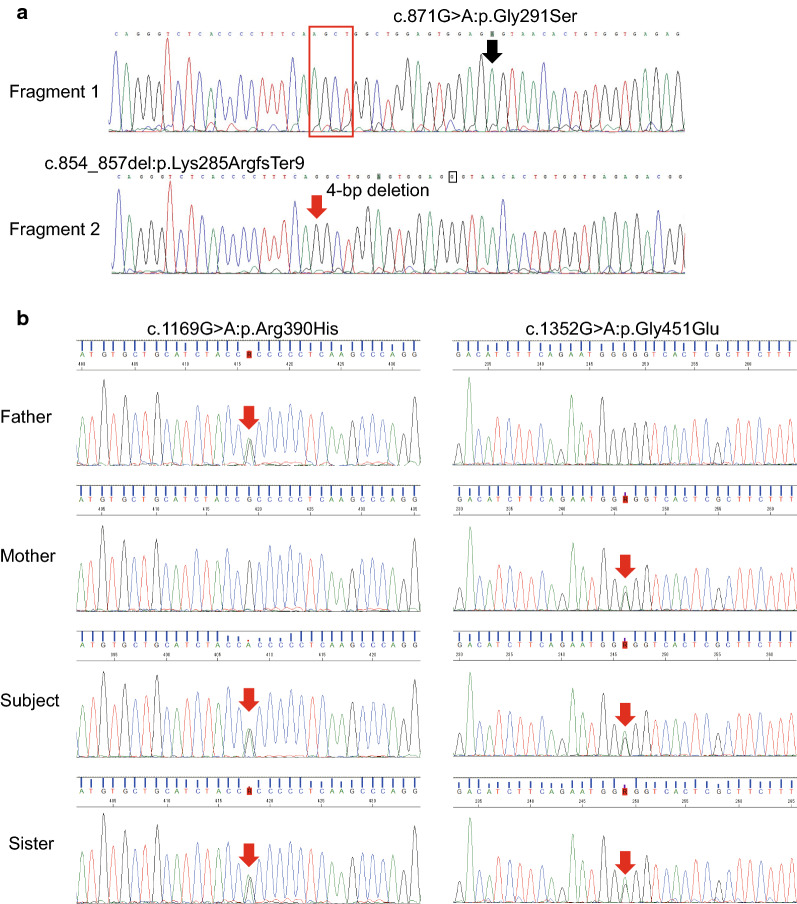
Genetic analysis of TRPV6 in THNP subjects. TA cloning followed by Sanger sequencing was performed for **a** subject 1 and **b** subject 2 with transient neonatal hyperparathyroidism (THNP). Novel compound heterozygous mutations were found in both subjects

*Subject 2* This male subject was born to a 29-year-old G0P0 mother of Japanese descent. The father was 30 years old and also of Japanese descent. The couple was healthy and non-consanguineous. They had a younger daughter who had the same genotype as the subject, but she did not have any complications or skeletal abnormalities (Fig. 1b). Fetal ultrasound detected no fetal abnormalities. The subject was delivered vaginally at 38 weeks of gestation. The birth weight was 2.805 kg (43 cm height, 35 cm OFC). Apgar scores were 7, 7 at 1 and 5 min, respectively. The child cried at birth but experienced respiratory distress 2 h after birth. The SpO_2_ was < 90% and the subject required nasal continuous positive airway pressure and oxygen for 6 and 8 days, respectively.

Radiographs taken on day 0 showed a bell-shaped thorax, femoral bending and proximal metaphyseal dysplasia that was possibly accompanied by metaphyseal fracture. Blood tests indicated hypocalcemia, extremely high PTH (high sensitive PTH was > 10,000 at day 0, iPTH was 90.8 pg/ml at day 16), and slightly depressed levels of 25(OH)-vitamin D. The subject was treated with calcium gluconate on day 0 and by day 1 the hypocalcemia had improved. Intact PTH levels were normalized on day 72. Bone symptoms had nearly resolved when the subject was 2 months old. Blood ALP levels were increased on day 0, peaked on day 72, and normalized at around 17 months. Femoral bending normalized at around 21 months.

### Sanger sequencing

All exonic regions of *TRPV6* (RefSeq ID: NM_018646.5, NT_007933.16) were amplified by PCR using QuickTaq HS (Toyobo, Japan) according to the manufacturer’s instructions. The PCR conditions were: 94 °C, 2 min followed by 35 cycles of 94 °C, 30 s; 62 °C, 30 s; 68 °C, 30 s (for exons 1–3 and 7–15) or 94 °C followed by 2 min and 35 cycles of 94 °C, 30 s; 55 °C, 30 s; 68 °C, 30 s (for exons 4–6). Primer sequences are listed in Additional file 1: Table S1.

### TA cloning for subject 1

PCR products were ligated into the TOPO vector using a TOPO TA cloning kit (Invitrogen, USA). After transformation of the vectors into *E. coli*, the resulting colonies that formed on a selection plate were subjected to colony PCR to screen for the presence of the insert using M13 forward and reverse primers. Colonies having the desired sequence were cultivated in LB medium supplemented with antibiotics. Plasmid isolation was performed using a QIAprep Spin miniprep kit (Qiagen, USA) and sequences were verified by Sanger sequencing.

### Mutagenesis

Site-directed mutagenesis was performed as described previously [8]. PCR was performed in a solution containing PrimeSTAR Max (TaKaRa, Japan), forward (5′-GGAGTGGAGAGTAACACTGTGATGTTTCAG) and reverse (5′-ATCACAGTGTTACTCTCCACTCCAGCCAGC) primers, and TRPV6/pcDNA3.1 (+) or TRPV6-2myc-N/pcDNA3.1(+) plasmid. The PCR conditions were: 30 cycles of 98 °C, 10 s; 55 °C, 15 s; 72 °C, 40 s.

### Cell culture and transfection

HEK293T cells were maintained and transfected with a plasmid carrying human TRPV6 as previously described [8, 9]. Briefly, cells were maintained in DMEM containing heat-inactivated FBS (10%), penicillin/streptomycin (100 units/ml), and GlutaMAX (Thermo Fisher, USA) with incubation in 5% CO_2_ at 37 °C. For transfection, Lipofectamine reagent (#18324010, Life Technologies Inc., USA) was used according to the manufacturer’s instructions. HEK293 cells were transfected with TRPV6 plasmid together with pGL1 and pCMV-DsRed-Express plasmid (0.1 µg) for patch-clamp and intracellular Ca^2+^ measurements, respectively. Transfected cells were identified by EGFP (patch-clamp) or DsRed (intracellular Ca^2+^ measurement) fluorescence.

### Electrophysiology

Whole-cell patch-clamp experiments were carried out 20–24 h after cell transfection as described previously with some modifications [8]. The time course used was based on difficulties associated with securing a giga-ohm seal under longer time courses. The standard bath solution contained 143 mM NaCl, 5 mM KCl, 2 mM CaCl_2_, 2 mM MgCl_2_, 10 mM glucose, 5 mM HEPES (pH 7.4). The divalent cation-free (DVF) bath solution contained 148 mM NaCl, 5 mM KCl, 10 mM glucose, 5 mM HEPES (pH 7.4). The NMDG bath solution contained 148 mM NMDG, 5 mM KCl, 10 mM glucose, 5 mM HEPES (pH 7.4 by HCl). The high calcium bath solution (30 Ca) contained 113 mM NMDG, 30 mM CaCl_2_, 2 mM MgCl_2_, 10 mM glucose, and 5 mM HEPES (pH 7.4 by HCl). The average osmolality of these solutions was 295 ± 4 mOsm. The pipette solution contained 140 mM CsCl, 2 mM MgCl_2_, 5 mM EGTA, and 10 mM HEPES (pH 7.3). Data were acquired using an Axopatch 200B amplifier (Axon Instruments, USA), digitized by Digidata 1440A (Axon Instruments, USA) at a 10 kHz sampling rate, and analyzed using pCLAMP10 software (Axon Instruments, USA). The membrane potential was clamped at − 60 mV. Voltage ramp pulses from − 100 to +100 mV (400 ms) were applied every 5 s. All recordings were performed at room temperature.

### Plasma membrane protein biotinylation and western blotting

Biotinylation and western blotting were performed 27–28 h after initiating transfection as previously described [8]. Transfected cells were washed with PBS and incubated with 0.5 mg/ml EZ-link-NHS-LC-biotin (Abcam, USA) for 10 min at 37 °C in a CO_2_ incubator. The EZ-link-NHS-LC-biotin solution was again added and incubated for 10 min at 37 °C. The biotinylation reaction was stopped by adding quenching buffer (100 mM glycine in PBS, pH 7.3) before the cells were transferred from the dish by pipetting. Cells were collected by centrifugation (12,000 rpm for 5 min at 4 °C) and stored at − 20 °C until use. Isolation of biotinylated proteins and western blotting was performed as described previously [8].

### Fura-2 Ca^2+^-imaging

Measurement of intracellular Ca^2+^ concentrations was conducted 20–24 h after starting the transfection as reported previously [8]. Cells were incubated with Fura-2-AM (5 µM, Life Technologies Inc., USA) at 37 °C for 1 h. The intracellular Ca^2+^ concentration was analyzed first in the standard bath solution and then in the DVF bath solution before superfusion of the 30 Ca bath solution. Ratiometric imaging was performed at 340 and 380 nm, and emission at 510 nm was recorded with a CCD camera (CoolSnap ES, Roper Scientific/Photometrics, USA) every 4 s. The F340/380 ratio was calculated using IP Lab software (Scanalytics Inc., USA) and data were analyzed using ImageJ (NIH).

## Results

**Fig. 2:**
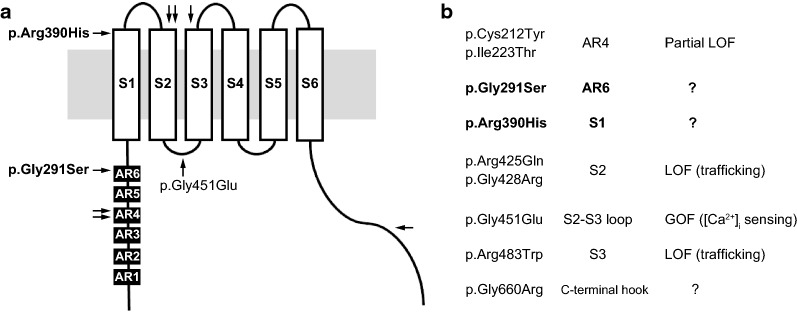
Distribution of TRPV6 mutations. *Arrows* indicate the reported mutations in THNP. Mutation hot spots are in the fourth ankyrin repeat domain (AR4) (*filled square*) and second transmembrane domain (S2) (*open square*). The novel mutations, pGly291Ser and p.Arg390His, localize to the AR6 and S1 domain, respectively. The p.Gly451Glu mutation is found on the S2–S3 loop, which is reported to play a role in [Ca^2+^]_i_-dependent inactivation [11]

Here, we report on two Japanese subjects with transient neonatal hyperparathyroidism (TNHP) with bone abnormalities. Both had pre- and post-natal history of skeletal abnormalities as well as elevated parathyroid hormone (PTH) and alkaline phosphatase levels (Table 1); these abnormalities gradually improved over time as previously reported [8]. Sanger sequencing of the TRPV6 gene identified the compound heterozygous mutations [NM_018646.5:c.854-857del:p.Lys285ArgfsTer9];[c.871G>A:p.Gly291Ser] in subject 1 (Fig. 1a) and [c.1169G > A:p.Arg390His];[c.1352G>A:p.Gly451Glu] in subject 2 (Fig. 1b). A novel mutation, pGly291Ser, was found in the sixth ankyrin repeat (AR) domain, whereas p.Arg390His localized to the outer edge of the first transmembrane (S1) domain [10]. These mutations were found far from previously reported mutation hotspots (Fig. 2). Meanwhile, the mutation p.Lys285ArgfsTer9 caused a frameshift that generates a truncation protein that lacks the transmembrane domains. As no genetic analysis was available for the parents, we performed TA cloning and confirmed that these were in fact compound heterozygous mutations (Fig. 1a).

**Table 1:** Pre- and post-natal clinical findings in two subjects with THNP associated with TRPV6 gene mutations

	Maternal/paternal age (years)	Prenatal ultrasound findings	GA at delivery (weeks)	Birth wt/lg/OFC (percentile)	Ionized calcium mmol/L (1.1–1.3)	Phosphorus mmol/L (1.6–2.6)	PTH pmol/L (1–6.9)	25OH vitamin D nmol/L (75–200)	Maternal 25OH vitamin D nmol/L (75–200)	Thorax hypoplasia and Respiratory dysfunction	Age at complete resolution of skeletal abnormality
Subject 1 (male)	39/44	Short, bell-shaped chest; short ribs, polyhydramnios	36	< 3rd/< 3rd^/^97th, > 95th	1.01	1.90	145.4	30.0	22.5	Yes	Still abnormal but substantial improvement
Subject 2 (male)	29/30	No abnormalities detected	38	< 3rd/40–50rd^/^92nd, > 90th	0.92	2.00	9.7	34.9	108.8 (1.4 mo)	Yes	1.8 years

Wt, weight; GA, gestational age; Wks, Weeks; OFC, head circumference; lg, length

**Fig. 3:**
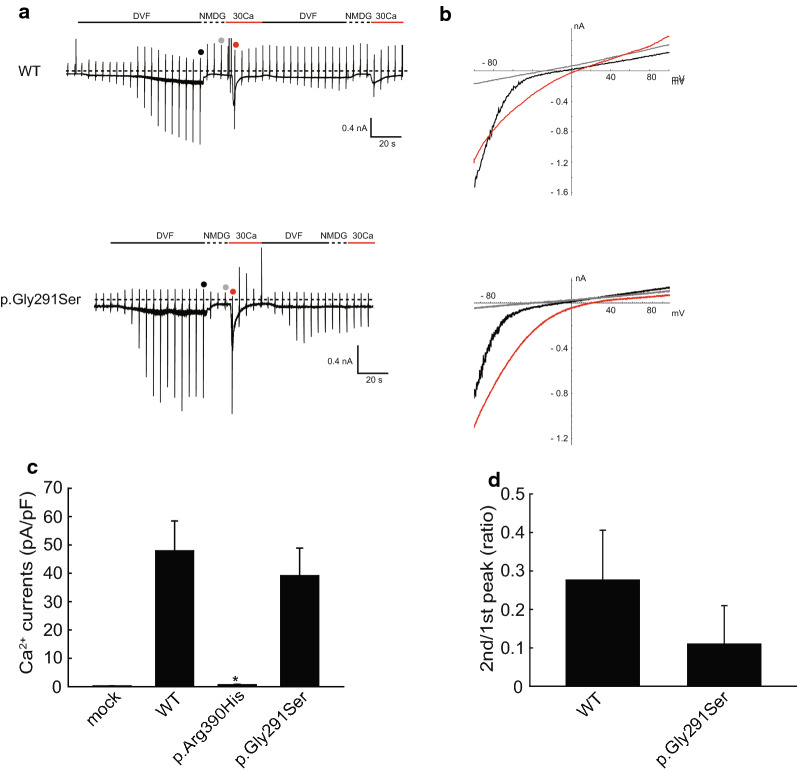
Whole-cell patch-clamp recordings in TRPV6-expressing HEK293T cells. **a** Representative time trace of membrane currents from cells expressing WT or p.Gly291Ser with a − 60 mV holding potential. Divalent cation-free (DVF) bath solution-evoked sustained currents and 30 mM Ca^2+^-evoked transient currents were observed in both traces. **b** Current–voltage relationship currents in **a**. *Red, gray and black* dots in the traces indicate the points at which I–V curves were generated. **c** Statistical analysis of the peak amplitude of Ca^2+^-evoked currents. The currents for p.Arg390His were significantly smaller compared to those for WT (*p* = 0.013, *n* = 4, Mann–Whitney rank-sum test). Currents for p.Gly291Ser did not significantly differ from that for the wild type

To analyze the functional significance of the p.Gly291Ser and p.Arg390His mutations, we transfected HEK293T cells with expression vectors carrying mutated TRPV6 and performed whole-cell patch-clamp recordings 20–24 h after transfection. HEK293T cells expressing the p.Gly291Ser mutant exhibited currents evoked in response to both divalent cation-free (DVF) and high Ca^2^ bath solution that were similar to that seen for WT (Fig. 3a, c). The amplitudes of the Ca^2+^-evoked currents were larger than those previously reported [8], most likely because of differences in the NMDG solution used. Here, we used Ca^2+^- and Mg^2+^-free NMDG solution in order to observe Ca^2+^ currents more clearly. Neither the current–voltage relationship nor reversal potential differed between WT and Gly291Ser (Fig. 3b), indicating a similar ion selectivity. We also found no significant difference in intracellular Ca^2+^-dependent inactivation, at least in our patch-clamp recordings within the time range of 20–30 s (Fig. 3d) [11]. These results suggested that apparent channel properties were preserved for the Gly291Ser mutation. In contrast, in cells expressing p.Arg390His no currents were evoked by DVF- or high Ca^2+^ bath solutions (Fig. 3c, Additional file 2: Fig. S1).

**Fig. 4:**
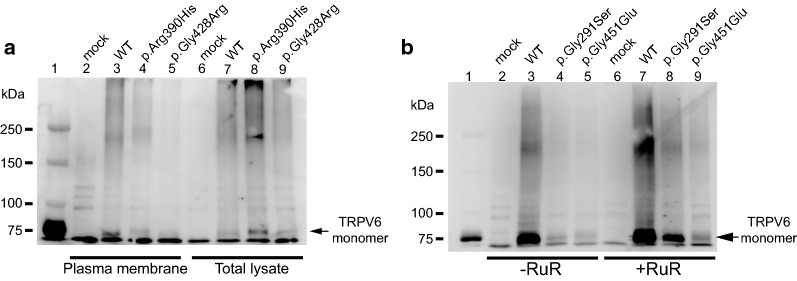
Western blot analysis of TRPV6 mutants. **a** Localization of TRPV6 proteins. Plasma membrane proteins in TRPV6-transfected HEK293T cells were biotinylated and collected with streptavidin beads. Myc-TRPV6 proteins were analyzed by western blotting using anti c-myc antibody (*left*). An ~ 80-kDa band was observed for cells expressing WT TRPV6 but not the p.Arg390His or p.Gly428Arg mutants. Bands for WT, p.Arg390His, and p.Gly428Arg were observed in total lysate (*right*), suggesting that the mutants had impaired trafficking to the plasma membrane. **b** Conventional western blotting of total cell lysates. Signals for p.Gly291Ser and p.Gly451Glu were significantly lower than that for WT in cells not treated with ruthenium red. In the presence of ruthenium red (4 µM) the signals were recovered, suggesting that cells expressing the mutants had Ca^2+^ overload

**Fig. 5:**
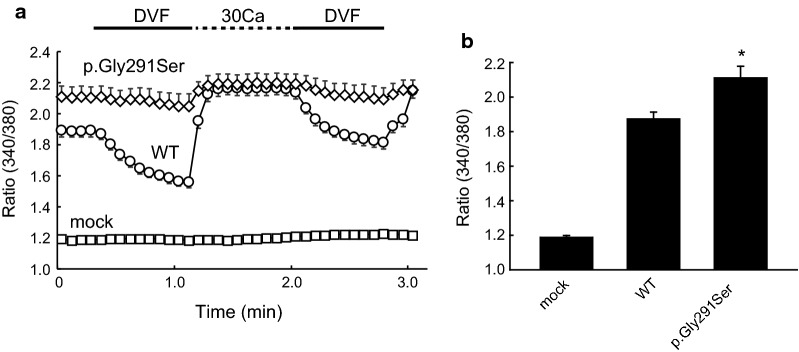
Measurement of intracellular Ca^2+^ concentration [Ca^2+^]_i_. **a** Time course of intracellular Ca^2+^ levels in WT- or pGly291Ser-expressing HEK293T cells. An increase in Ca^2+^ level after application of 30 mM Ca^2+^, an indication of plasma membrane Ca^2+^ permeability, was observed for WT cells. In cells expressing p.Gly291Ser, the Ca^2+^ level was significantly higher than that for WT in the steady state (2 mM Ca^2+^, time = 0; *p* = 0.012 vs. WT, *n* = 39–49, Mann–Whitney rank-sum test), and the level did not increase substantially upon application of 30 mM Ca^2+^, most likely due to saturation. These results suggest a Ca^2+^ overload in the steady state

Thus, we hypothesized that trafficking of p.Arg390His proteins was impaired. Indeed, similar to the TRPV6 S2 transmembrane domain mutant p.Gly428Arg that was previously shown to have deficient trafficking, p.Arg390His did not localize to the plasma membrane, but was present in total cell lysates (Fig. 4a). Meanwhile, for the TRPV6 AR6 mutant p.Gly291Ser, we could not detect the presence of this protein even with longer transfection time, which is similar to that previously reported for p.Gly451Glu. With a shorter transfection time, we could detect it in the plasma membrane (Additional file 3: Fig. S2). We thus hypothesized that the p.Gly291Ser mutation affects intracellular Ca^2+^-dependent inactivation over a longer time range as was observed previously [8]. To examine the effects of Ca^2+^ overload and cell death, we performed conventional western blotting of cell lysates from p.Gly291Ser-transfected cells with or without treatment with the TRP channel blocker ruthenium red (RuR). The pGly291Ser proteins were detectable in RuR-treated cells, suggesting that impaired [Ca^2+^]_i_-dependent inactivation over a longer time range leads to Ca^2+^ overload and cell death (Fig. 4b). In cells without RuR treatment the band for p.Gly291Ser was faint, possibly because this mutant could elicit Ca^2+^ overload leading to detachment of cells from the culture dish. In the presence of RuR, p.Gly291Ser TRPV6-derived Ca^2+^ influx is blocked and as such no Ca^2+^ overload would be elicited. Upon measurement of intracellular Ca^2+^ levels with Fura-2, we observed a significantly higher Ca^2+^ concentration in cells expressing p.Gly291Ser under a steady-state condition *(p* < 0.05, n = 39–49, Mann–Whitney test, Fig. 5a, b). Moreover, the intracellular Ca^2+^ in these cells did not change significantly following superfusion of divalent cation-free solution (DVF) or 30 Ca bath solution, likely because the Ca^2+^ concentration was nearly saturated. These results supported our hypothesis that the Gly291Ser mutation causes impaired [Ca^2+^]_i_-dependent inactivation across a longer time range that may be too long to allow detection in patch-clamp recordings.

## Discussion

Transient receptor potential vanilloid 6 (TRPV6) is a member of the TRP channel family that has very high Ca^2+^-selectivity [12]. TRPV6 is reported to play a role in Ca^2+^ absorption in the intestine [13, 14] and the epididymis [15], as well as in maternal–fetal Ca^2+^ transport across the placenta [16], suggesting a role for this channel in transcellular Ca^2+^ transport in particular organs and tissues [12, 17]. Despite its physiological significance, the molecular mechanisms that regulate this channel remain largely undefined. For example, how TRPV6 maintains intracellular Ca^2+^ concentrations that in turn maintain continuous transcellular Ca^2+^ transport is unclear.

Previously, we found that mutations in TRPV6 cause transient neonatal hyperparathyroidism (TNHP) with bone abnormalities [8]. These mutations localize to hotspots on the extracellular side of S2 and S3 transmembrane domains, as well as to the intracellular ankyrin repeat domain 4 (AR4). Functional analysis indicated that: (1) TRPV6 with mutations in the transmembrane domains exhibit impaired trafficking to the plasma membrane; (2) mutations in AR4 are associated with partial loss-of-function, likely due to protein instability; and (3) the pGly451Glu mutation leads to intracellular Ca^2+^ overload due to impaired [Ca^2+^]_i_-dependent inactivation [8]. Another study identified the C-terminal TRPV6 mutation p.Gly660Arg, although the functional significance of this mutation is unclear [18].

In this study, we identified novel TRPV6 mutations in two patients with TNHP: p.Gly291Ser (subject 1), and p.Arg390His (subject 2). The results of the present study suggested that pGly291Ser might cause intracellular Ca^2+^ overload. In terms of [Ca^2+^]_i_-dependent inactivation, in our patch-clamp recordings we found no significant difference between the mutant and WT upon repetitive application of calcium over 20–30 s. This outcome is a general indicator for the [Ca^2+^]_i_-dependent fast/intermediate inactivation. Meanwhile, the intracellular Ca^2+^ concentration was significantly higher in the mutant-expressing cells than in cells expressing WT. Although the reason for this difference is unclear, Ca^2+^-selective channels can be inhibited via multiple pathways, including those having fast and slow inactivation kinetics [11]. Based on our results, we propose that p.Gly291Ser has slower inactivation than can be observed in the repetitive application time range.

In subject 1, another allele carried a frameshift mutation (Lys285ArgfsTer9), suggesting that pGly291Ser expression could be upregulated by a decrease in maternal–fetal Ca^2+^ transport caused by a frameshift mutation into another allele, thus leading to further Ca^2+^ overload and cell death as suggested in the previous report [8]. Subject 2 had the mutation p.Arg390His and demonstrated impaired maternal–fetal Ca^2+^ transport, and also had upregulation of another TRPV6 allele, p.Gly451Glu, that leads to a Ca^2+^ overload [8]. These two examples strongly suggest that, in many cases of THNP, a combination of mutations can dysregulate intracellular Ca^2+^ homeostasis. Maintenance of intracellular Ca^2+^ concentration is highly important to maintain transcellular Ca^2+^ transport. Further studies will be essential to reveal the detailed mechanism by which the AR6 domain in particular regulates TRPV6 activity. Knock-in mice carrying these disease mutations would be valuable to analyze the regulation of transcellular Ca^2+^ transport in vivo.

Although Arg390 lies on the outer edge of the transmembrane domain, it is nonetheless far from previously described mutation hot spots. This location might be related to the normocalcemic phenotype exhibited by subject 2 relative to that seen for subjects 4 and 5 in the previous report that both carried p.Gly451Glu and that were both hypocalcemic at birth. Indeed, a sister of subject 2 having the same genotype was not diagnosed with TNHP. *Trpv6* KO mice exhibit a reduced intestinal and placental calcium transport, strongly suggesting that *Trpv*6 is critical for total body Ca^2+^ homeostasis [13, 14, 16]. However, in these same *Trpv*6 knockout mice, results of several reports indicated that *Trpv*6 is not crucial for 1,25(OH)_2_-vitamin D_3_-dependent intestinal Ca^2+^ absorption [19]. We propose that these different phenotypes could be due to different Ca^2+^ environments such as differences in the Ca^2+^ content of drinking water or maternal vitamin D levels. In the case of calcium intake, in a 2017 study Japanese subjects were reported to have an average daily Ca^2+^ intake of 517 mg. Women aged 20–29 and those aged 30–39 consumed an average of 420 mg/day and 421 mg/day, respectively. These amounts were smaller than those for Western countries where the consumption of dairy products is generally higher [20]. Based on these findings, for subject 2 who carried p.Arg390His and had THNP, whereas a sister having the same genotype did not, we hypothesize that the Ca^2+^ status of the mother might have differed between pregnancies. If this is indeed the case, there could be other potential mutations/polymorphisms that have milder effects than p.Arg390His, and would lead to TNHP depending on the Ca^2+^ status of the mother. In other words, there may be a spectrum of transient neonatal hyperparathyroidism that is affected by both genotype and environment. Rare variants as well as common SNPs in the TRPV6 gene should be further analyzed in different human populations to determine the relationship between TRPV6 variants and disease severity, and reveal the molecular mechanism by which TRPV6 activity is regulated as well as the significance of molecular evolution of the TRPV6 gene [21–23].

## Conclusion

Mutations in the gene encoding the TRP channel TRPV6 cause transient neonatal hyperparathyroidism (TNHP). In this study, we report novel mutations in the TRPV6 S1 and AR6 domains that occurred outside of domains recognized as being mutation hotspots (e.g., S2, S3, AR4 and the S2–S3 loop). Unexpectedly, we found that the mutation in AR6 was associated with increased intracellular Ca^2+^ concentrations. This result suggests that the AR6 domain of TRPV6 is involved in the maintenance of the [Ca^2+^]_i_ homeostasis that is crucial for sustained, unidirectional Ca^2+^ transport.

### Supplementary information

**Additional file 1: Table S1.** Oligonucleotide sequences of primers used for Sanger sequencing of the human TRPV6 gene.

**Additional file 2: Figure S1.** Representative time trace of whole cell currents from HEK293T cells expressing WT or p.Arg390His-TRPV6 at a − 60 mV holding potential. Dashed lines indicate zero current level. Whole-cell patch-clamp recordings were carried out 22–24 hours after the transfection using standard bath solution (143 mM NaCl, 5 mM KCl, 1 mM CaCl2, 2 mM MgCl2, 5 mM HEPES, 10 mM glucose), divalent-free (DVF) solution (148 mM NaCl, 5 mM KCl, 5 mM HEPES, 10 mM glucose), NMDG solution (149 mM NMDG, 1 mM CaCl2, 2 mM Mg Cl2, 5 mM HEPES, 10 mM glucose), or 30 mM calcium solution (113 mM NMDG, 30 mM CaCl2, 2 mM MgCl2, 5 mM HEPES, 10 mM glucose), and pipette solution containing 100 mM Cs-aspartate, 40 mM CsCl, 1 mM MgCl2, 10 mM EGTA, 5 mM HEPES (pH 7.2 with CsOH). Osmolarity was confirmed to be ~290 mOSm/kg. Data were sampled using an Axopatch 200B amplifier and pCLAMP software (Axon Instruments, USA). Membrane potential was clamped at − 60 mV.

**Additional file 3: Figure S2.** Localization of TRPV6 proteins in the plasma membrane. Plasma membrane proteins of transfected HEK293 cells were biotinylated, collected with streptavidin beads. Myc-TRPV6 proteins were analyzed by Western blotting using anti c-myc antibody. The 80 kDa band was observed for cells expressing WT and p.Gly291Ser. Anti-Na+-K+-ATPase antibody was used for the loading controls. Plasma membrane protein biotinylation were carried out 22 hours after initiating transfection. Transfected cells were incubated twice with 0.5 mg/ml EZ-link-NHS-LC-biotin (Abcam, USA) at 37 ºC for 10 min. The biotinylation was stopped with quenching buffer (100 mM glycine in PBS, pH 7.3) before the cells were from the dish by adding the lysis buffer [10 mM Tris-HCl (pH 7.2), 150 mM NaCl, 1 mM EDTA, 1 mM Na3VO4, 1% NP-40, 1 × Complete protease inhibitor cocktail (Sigma, USA)]. Biotinylated proteins were collected by the magnetic beads (Dynabeads MyOne StreptT1, Thermofisher scientific, USA) under manufacture’s instruction. Western blotting was performed with 7.5 % TGX gel (Bio-rad, USA), and the signal was visualized by the Light capture system (AE-6981, ATTO, Japan). Anti-Na+,K+-ATPase antibody with HRP (EP1845Y, Abcam, USA) was used for the loading control with 1/1000 dilution.

## Data Availability

The datasets used and/or analyzed during the current study are available from the corresponding author on reasonable request.

## Abbreviations

TRPV6: Transient receptor potential subfamily V, member 6
PCR: Polymerase chain reaction
KO: Knockout
AR: Ankyrin repeat domain
DVF: Divalent cation-free solution
THNP: Transient neonatal hyperparathyroidism
