# Supplementary material for: Novel TRPV6 mutations in the spectrum of transient neonatal hyperparathyroidism
Source: J Physiol Sci. 2020 Jul 9;70:33. doi: 10.1186/s12576-020-00761-2 (PMC10717230; doi:10.1186/s12576-020-00761-2)
Supplement: Supplementary file 1 — Additional file 1: Table S1. Oligonucleotide sequences of primers used for Sanger sequencing of the human TRPV6 gene. [file 12576_2020_761_MOESM1_ESM.pdf]

Primer name	5'→3'
**TRPV6 ex1F**	GTCCTGGCTGGCTCTGCCAAGTG
**TRPV6 ex1R**	CTGGGAGCACCATCTGTCCAG
**TRPV6 ex2-3F**	CTGAGGTTCCTGCTGCTGAG
**TRPV6 ex2-3R**	GATGGGCAGTCAGGACTGAC
**TRPV6 ex4-6F**	GTTCTCCATCCCAGGCTACC
**TRPV6 ex4-6R**	GCATGGGATCTAGACTCTG
**TRPV6 ex7-8F**	CGTCCTCCTGCACATGCATC
**TRPV6 ex7-8R**	CCAGGGTCACCAGTCTTAGC
**TRPV6 ex9-10F**	GCACCATGGAAATGCAGGG
**TRPV6 ex9-10R**	GGCTCTGGAGCTAAGGTTC
**TRPV6 ex11F**	GGCTGAATCTGCATGTGTGGG
**TRPV6 ex11R**	CCTGACCTGCTTTGGTGTGG
**TRPV6 ex12F**	CCTGTTAGAGGCTGGATGGG
**TRPV6 ex12R**	CATCCTAAGAGCCACCTCTCC
**TRPV6 ex13F**	CAGGATGGACATGCACGCAG
**TRPV6 ex13R**	CTGTCTCTCAGCTTGGCAGG
**TRPV6 ex14F**	GCTGCGTCATGGATGTTGGGTC
**TRPV6 ex14R**	GCATAGAGCCTAGGGAGCAC
**TRPV6 ex15F**	GCTTTCTGAGGCCTAGCTGC
**TRPV6 ex15R**	CTGGGAGATGAGACCTCTGG
