# Supplementary figures and images for: Novel TRPV6 mutations in the spectrum of transient neonatal hyperparathyroidism
Source: J Physiol Sci. 2020 Jul 9;70:33. doi: 10.1186/s12576-020-00761-2 (PMC10717230; doi:10.1186/s12576-020-00761-2)

Fig. S2

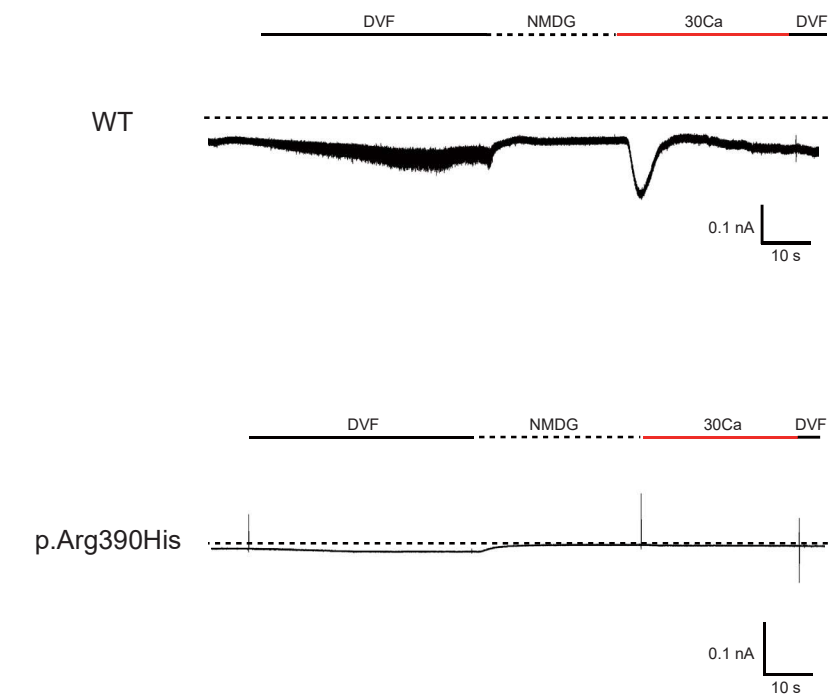

Supplement: Supplementary file 2 — Additional file 2: Figure S1. Representative time trace of whole cell currents from HEK293T cells expressing WT or p.Arg390His-TRPV6 at a − 60 mV holding potential. Dashed lines indicate zero current level. Whole-cell patch-clamp recordings were carried out 22–24 hours after the transfection using standard bath solution (143 mM NaCl, 5 mM KCl, 1 mM CaCl2, 2 mM MgCl2, 5 mM HEPES, 10 mM glucose), divalent-free (DVF) solution (148 mM NaCl, 5 mM KCl, 5 mM HEPES, 10 mM glucose), NMDG solution (149 mM NMDG, 1 mM CaCl2, 2 mM Mg Cl2, 5 mM HEPES, 10 mM glucose), or 30 mM calcium solution (113 mM NMDG, 30 mM CaCl2, 2 mM MgCl2, 5 mM HEPES, 10 mM glucose), and pipette solution containing 100 mM Cs-aspartate, 40 mM CsCl, 1 mM MgCl2, 10 mM EGTA, 5 mM HEPES (pH 7.2 with CsOH). Osmolarity was confirmed to be ~290 mOSm/kg. Data were sampled using an Axopatch 200B amplifier and pCLAMP software (Axon Instruments, USA). Membrane potential was clamped at − 60 mV. [file 12576_2020_761_MOESM2_ESM.pdf]

Fig. S3

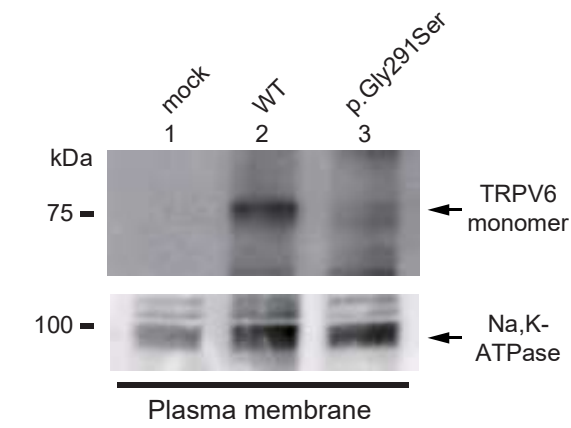

Supplement: Supplementary file 3 — Additional file 3: Figure S2. Localization of TRPV6 proteins in the plasma membrane. Plasma membrane proteins of transfected HEK293 cells were biotinylated, collected with streptavidin beads. Myc-TRPV6 proteins were analyzed by Western blotting using anti c-myc antibody. The 80 kDa band was observed for cells expressing WT and p.Gly291Ser. Anti-Na+-K+-ATPase antibody was used for the loading controls. Plasma membrane protein biotinylation were carried out 22 hours after initiating transfection. Transfected cells were incubated twice with 0.5 mg/ml EZ-link-NHS-LC-biotin (Abcam, USA) at 37 ºC for 10 min. The biotinylation was stopped with quenching buffer (100 mM glycine in PBS, pH 7.3) before the cells were from the dish by adding the lysis buffer [10 mM Tris-HCl (pH 7.2), 150 mM NaCl, 1 mM EDTA, 1 mM Na3VO4, 1% NP-40, 1 × Complete protease inhibitor cocktail (Sigma, USA)]. Biotinylated proteins were collected by the magnetic beads (Dynabeads MyOne StreptT1, Thermofisher scientific, USA) under manufacture’s instruction. Western blotting was performed with 7.5 % TGX gel (Bio-rad, USA), and the signal was visualized by the Light capture system (AE-6981, ATTO, Japan). Anti-Na+,K+-ATPase antibody with HRP (EP1845Y, Abcam, USA) was used for the loading control with 1/1000 dilution. [file 12576_2020_761_MOESM3_ESM.pdf]
